# Central Insulin-Like Growth Factor-1-Induced Anxiolytic and Antidepressant Effects in a Rat Model of Sporadic Alzheimer’s Disease Are Associated with the Peripheral Suppression of Inflammation

**DOI:** 10.3390/cells14151189

**Published:** 2025-08-01

**Authors:** Joanna Dunacka, Beata Grembecka, Danuta Wrona

**Affiliations:** Department of Animal and Human Physiology, Faculty of Biology, University of Gdansk, Wita Stwosza 59 Street, 80-308 Gdansk, Poland; joanna.dunacka@ug.edu.pl (J.D.); beata.grembecka@ug.edu.pl (B.G.)

**Keywords:** sporadic Alzheimer’s disease model, insulin-like growth factor-1, anhedonia, anxiety, peripheral inflammation

## Abstract

(1) Insulin-like growth factor-1 (IGF-1) is a neurotrophin with anti-inflammatory properties. Neuroinflammation and stress activate peripheral immune mechanisms, which may contribute to the development of depression and anxiety in sporadic Alzheimer’s disease (sAD). This study aims to evaluate whether intracerebroventricular (ICV) premedication with IGF-1 in a rat model of streptozotocin (STZ)-induced neuroinflammation can prevent the emergence of anhedonia and anxiety-like behavior by impacting the peripheral inflammatory responses. (2) Male Wistar rats were subjected to double ICVSTZ (total dose: 3 mg/kg) and ICVIGF-1 injections (total dose: 2 µg). We analyzed the level of anhedonia (sucrose preference), anxiety (elevated plus maze), peripheral inflammation (hematological and cytometric measurement of leukocyte populations, interleukin (IL)-6), and corticosterone concentration at 7 (very early stage, VES), 45 (early stage, ES), and 90 days after STZ injections (late stage, LS). (3) We found that ICVIGF-1 administration reduces behavioral symptoms: anhedonia (ES and LS) and anxiety (VES, ES), and peripheral inflammation: number of leukocytes, lymphocytes, T lymphocytes, monocytes, granulocytes, IL-6, and corticosterone concentration (LS) in the rat model of sAD. (4) The obtained results demonstrate beneficial effects of central IGF-1 administration on neuropsychiatric symptoms and peripheral immune system activation during disease progression in the rat model of sAD.

## 1. Introduction

Alzheimer’s disease (AD) is a progressive neurodegenerative disorder. The disease occurs in two forms: familial (fAD) and sporadic (sAD). The second type (also known as late onset) is diagnosed much more frequently, as it affects up to 95% of cases and its occurrence relates to the elderly, i.e., over 65 years of age [1]. The exact causes of the development of sAD are not known, but the following aspects, among other things, are suspected: genetic risk factors (ε4 form of *APOE gene*), dysfunction of insulin pathways and glymphatic system in the brain, β-amyloid accumulation, the presence of oxidative stress, neuroinflammation, chronic stress, head trauma, sleep disturbances, exposure to aluminum, malnutrition, and virus infection [1,2,3,4,5,6,7,8,9]. The disorder is characterized by impaired glucose metabolism, mitochondrial dysfunction, neuroinflammation, the presence of β-amyloid (Aβ) and tau aggregation in the brain, memory loss, anxiety, and depression, and hormonal changes, such as the dysregulation of the hypothalamic–pituitary–adrenal (HPA) axis [2,10,11]. Currently, the therapy of disease (drugs: memantine, donepezil, galantamine, and rivastigmine) is primarily based on increasing acetylcholine and reducing glutamate levels in the brain to ensure the proper functioning of cholinergic pathways and reduce neuronal death [12]. However, lecanemab is also used in this application. This substance is an antibody, directed against large soluble forms of beta-amyloid and insoluble fibrils that build senile plaques [13]. Therefore, the available treatment focuses only on selected symptoms of AD, which is the basis for the search for more drugs to ameliorate other symptoms of the disease.

One of the most common models of sAD is intracerebroventricular (ICV) injections of streptozotocin (STZ). The administration of this substance allows for the representation of symptoms and features of sAD in animals, such as neuroinflammation, oxidative stress [14], disturbances of insulin signaling in the brain, insulin-resistant brain state (IRBS) [15] changes in the activity of enzymes involved in cerebral glucose metabolism [16], and also behavioral disturbances related to spatial memory, anxiety, and anhedonia disorders [17,18,19,20,21]. Moreover, after the central injection of STZ, the accumulation of Aβ, building of senile plaques, and hyperphosphorylated tau protein forming neurofibrillary tangles were observed [22].

The presence of oxidative stress in the brain increases the activity of the HPA axis by reducing the function of its negative feedback loop [23]. One of the results of HPA axis activation is the secretion of glucocorticoids (GCs) [23], for which receptors are located on all types of immune cells [24]. Glucocorticoids are hormones that inhibit the immune response by inducing the apoptosis of immune cells or reducing the activity of NF-κB and MAPK pathways, thereby preventing the activation of genes that promote inflammation, but in some cases GCs increase immune response [25]. GCs inhibit neurogenesis, synaptic plasticity, and neuronal survival, and increase Aβ synthesis as well as tau protein hyperphosphorylation, and therefore support the development of AD [26]. Moreover, peripheral inflammation also affects the central nervous system by damaging the brain–blood barrier (BBB) and allowing peripheral immune cells to flow into the brain, which causes a stronger neuroimmunological response [27]. It was found that in the course of AD neutrophils accumulate near Aβ and disrupt blood flow in the brain, helper TCD4^+^ (Th17) lymphocytes synthesize interleukin-17 (IL-17), which contributes to cognitive impairment, T cytotoxic CD8^+^ lymphocytes disrupt synaptic plasticity, B lymphocytes increase the accumulation of senile plaques or microglial dysfunction, and NK cells, through interaction with microglia, contribute to the synthesis of inflammatory mediators [28]. Changes in the immune response also contribute to behavioral disorders such as anxiety or depression [29]; therefore, the neuroinflammation and peripheral immune system activation observed in AD may contribute to behavioral changes.

Insulin-like growth factor-1 (IGF-1) is a neurotrophin with anti-inflammatory properties. IGF-1 in the brain reduces the neuroroinflammatory response by promoting the conversion of microglia into an anti-inflammatory M2 phenotype and inhibits reactive oxygen species (ROS) production [30], thereby repairing damaged neural tissue [31]. Moreover, it can enhance neurogenesis in the hippocampus, protect neurons against Aβ-induced toxicity in the hippocampus, and increase the release of neurotransmitters, such as acetylcholine. Acetylcholine acts as an antioxidant, reduces the levels of pro-inflammatory cytokines and reactive species [32,33,34], and its synthesis is reduced in AD. In addition, IGF-1 reduces depressive disorders and memory impairment, and improves cognitive functions [32,35,36]. Furthermore, a higher level of IGF-1 can prevent neurodegeneration [37].

Although anxiety and depressive disorders are not major symptoms of sporadic Alzheimer’s disease and can occur as symptoms of other neurodegenerative disorders, they are an important aspect of treating the disorder. In patients with AD, the following behavioral changes were found: anxiety (39%), depression (42%), aggression (40%), and apathy (49%) [12]. This being the case, eliminating or reducing these negative symptoms of the disease is both a challenge and a real need for improving the quality of life of patients and their caregivers. Therefore, the aim of our study was to examine whether the intracerebroventricular administration of IGF-1 reduces anxiety and depressive disorders and influences peripheral inflammation in a rat model of sporadic Alzheimer’s disease, and whether this effect is dependent on the stage of the disease. To investigate as thoroughly as possible the effect of ICVIGF-1 administration, behavioral determinations (sucrose preference test, elevated plus maze) were carried out at four time points and peripheral immune parameters (hematological and cytometric distribution of leukocyte population; determination of IL-6, IL-10, and corticosterone concentrations) were measured at two time points.

## 2. Materials and Methods

### 2.1. Animals

**Figure 1:**
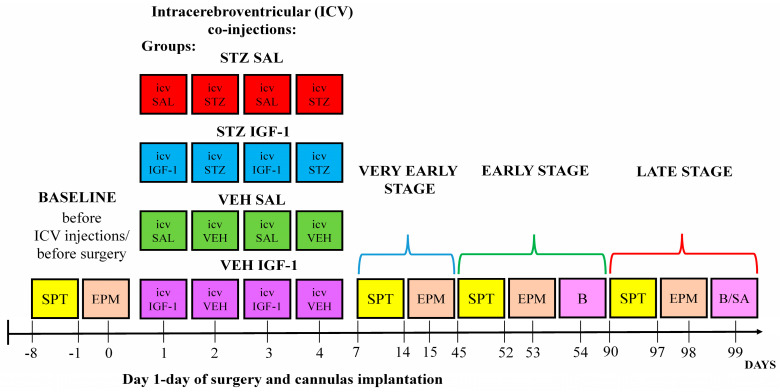
Scheme of procedures and group assignments. Explanations: SPT—sucrose preference test; EPM—elevated plus maze; ICVIGF-1—intracerebroventricular (ICV) injections of insulin-like growth factor-1 (IGF-1, total dose: 2 µg, divided into two injections on days 1 and 3, 0.5 µg/2 µL/ventricle, neurotrophin treatment); ICVSAL—intracerebroventricular (ICV) injections of saline (SAL, 2 µL/ventricle); ICVSTZ—intracerebroventricular (ICV) injections of streptozotocin (STZ, total dose: 3 mg/kg, divided into two injections on days 2 and 4, 0.75 mg/kg/2 µL/ ventricle, induction of sporadic Alzheimer’s disease model); ICVVEH—intracerebroventricular (ICV) injections of vehicle (VEH, 2 µL/ventricle), B—blood collection, B/SA—blood collection and sacrifice of animals; very early stage—stage of sporadic Alzheimer’s disease progression starting 7 days after ICV injections; early stage—stage of sporadic Alzheimer’s disease progression starting 45 days after ICV injections; and late stage—stage of sporadic Alzheimer’s disease progression starting 90 days after ICV injections.

Research was carried out in accordance with Directive 2010/63/EU of the European Parliament, and with the approval of the Local Ethical Committee for the Care and Use of Laboratory Animals at the University of Technology in Bydgoszcz, Poland (no. 60/2017). The experiment was conducted using male Wistar Han rats (N = 32, weight: 300 ± 10 g, and age: 11–12 weeks old) bred in the Tri-City Central Animal Laboratory, Research and Service Centre of the Medical University of Gdansk in Poland (breeder registration number: 041). All research procedures were performed at the Vivarium of the University of Gdańsk, Faculty of Biology (license number: 0169). Rats were housed with one individual per cage (width: 20 cm, length: 40 cm, and height: 18 cm), with visual and olfactory contact with other individuals, on a 12 h light/dark cycle (lights on at 06:00 a.m.) in an air-conditioned, constant-temperature (22 ± 2 °C) room, with constant (ad libitum) access to water and food. Before starting the measurements in basal conditions, animals were acclimatized to laboratory conditions (2 weeks) and then to the presence of experimenters (handling, 2 weeks, 5 min/day) in order to minimize the stress caused by the experimenters and procedures. After the completion of the baseline behavioral measurement and before stereotaxic cannula implantation, the animals were randomly divided into 4 groups (N = 8/group): STZ SAL, STZ IGF-1, VEH SAL, and VEH IGF-1, according to the diagram presented in Figure 1.

### 2.2. Induction of Sporadic Alzheimer’s Disease Model and Treatment with Insulin-Like Growth Factor-1 (IGF-1)

Briefly, the animals were subjected to inhalation anesthesia (2.5% isoflurane, airflow: 0.5 L/min) using a vaporizer (Rothacher-Medical, Heitenried, Switzerland) and pump (Bitmos OXY 6000, Bitmos GmbH, Dusseldorf, Germany), and afterward the head of the rat was fixed using a stereotaxic apparatus (Hugo Sachs Elektronik, March, Germany). During the surgeries, the guide cannulas (3 mm, C311G/Spc, Bilaney, Dusseldorf, Germany) were implanted bilaterally according to coordinates from the stereotaxic atlas [38] (coordinates: AP: −1.3 mm, L: ±2 mm). The cannulas were compatible with an injection needle (4 mm, C311I/Spc, Bilaney, Dusseldorf, Germany) and both constituted a route of administration of chemicals to lateral ventricles of the brain (D: +3.6 mm). The two guide cannulas were constantly anchored to three stainless steel skull screws by using a dental acrylic (Duracryl, spofa Dental a.s., Jicin, Czech Republic). ICV injections were carried out on days 1–4.

In order to induce the Alzheimer’s disease model, the ICV injections of streptozotocin (STZ, Sigma Aldrich, St. Louis, MO, USA, total dose: 3 mg/kg, 2 × 1.5 mg/kg, and 0.75 mg/kg/2 µL/ventricle) [18,19,20,36], dissolved in a 0.02 M citrate buffer (pH 4.5), were administered on days 2 and 4. Control rats were ICV injected with a citrate buffer (VEH, 2 µL/ventricle) (Figure S1).

On days 1 and 3, insulin-like growth factor-1 (IGF-1, Biotechne, 291-G1, Minneapolis, MN, USA) was administered ICV at a total dose of 2 µg (2 × 1 µg, 0.5 µg/2 µL/ventricle diluted in saline). The IGF-1 dose and route of administration were based on our [36] and other authors’ reports [35,39]. Rats from control groups received ICV saline (SAL) injections. A single 1 µg ICV injection of IGF-1, which was used in the present study, was sufficient for inducing improvements in behavioral activity related to anxiety and anhedonia, as indicated by the increased percentage of time in the open arms of the EPM test and the increased sucrose intake in the SPT in a rat model of ICVSTZ-induced sAD.

All co-injections (ICV) of IGF-1/SAL and STZ/VEH were carried out by using a microinfusion pump (Legato-100—Series Syringe Pump, KD SCIENTIFIC, Holliston, MA, USA) and a Hamilton syringe (10 μL) connected to an injection needle by a polyethylene tube. The rate of administration of substances was 0.5 µL/min. To prevent the backflow of the solution, the injection needle was left inside the guide cannula for an additional 1 min. After the surgery and co-injections were completed, the animals were moved to a warm room, where they stayed until they regained consciousness. Following ICV implantation of the cannulas and ICV injection of substances, the animals remained in their home cages for a 1-week recovery period until the wounds on their heads healed. The rats were then subjected to two behavioral tests.

### 2.3. Sucrose Preference Test (SPT)

In order to determine the level of anhedonia, a primary depression-like behavior, the sucrose consumption in the sucrose preference test (SPT) was measured according to the procedure described previously [40,41], with some modifications. The SPT was performed at the baseline and at the very early (starting 7 days after the induction of sAD, VES), early (starting 45 days after the induction of sAD, ES), and late (starting 90 days after the induction of sAD, LS) stages of sAD progression. In order to determine the sucrose preference coefficient, the animals had access to two bottles for 8 consecutive days (7 measurements). The first bottle always contained drinking water, while the second bottle contained an aqueous solution of sucrose, with the first three measurements concerning a 2% solution and the last four measurements concerning a 4% sucrose solution. All measurements were collected every 24 h, as there were differences in the weight of the bottle with liquid. Additionally, the position of the bottles was changed after 24 h to eliminate side preferences by the rats. The data obtained during the last measurement, in which the sucrose concentration was 4%, were presented as a percentage of sucrose solution consumption relative to all fluid consumption over 24 h. The remaining days of the test were treated as a training phase.

Sucrose consumption preference was calculated as follows:

$$\mathrm{sucrose}\;\mathrm{preference}\;\left(\%\right)=\frac{\mathrm{sucrose}\;\mathrm{intake}\;(g)}{\left(\mathrm{water}\;+\;\mathrm{sucrose}\;\mathrm{intake}\;\left(g\right)\right)}\;\times \;100$$

The trained observer who was blinded to the experimental treatment of the animals analyzed the behavioral responses.

### 2.4. Elevated Plus Maze (EPM)

In order to determine the level of anxiety, the time spent, entrances, and episodes of miction and defecation in open arms, center and closed arms of the elevated plus maze (EPM) were measured. The test was performed according to a methodology described earlier [20,42], with some modifications. The EPM apparatus was made of plexiglass and consisted of two black, open arms (50 × 10 cm) and two opposite black, closed arms (high walls: 40 cm) connected by a black square center (10 × 10 cm), and thus the maze formed a plus sign. The whole maze was elevated 50 cm above the level of the floor. All EPM trials (duration: 5 min) were recorded using a video camera (200 cm above the apparatus) connected to a video-tracking system (EthoVision XT, Noldus, Wageningen, The Netherlands), and were conducted between 8:00 and 12:00 a.m. To ensure reproducible measurements between the baseline conditions and disease stages (a. very early stage, VES, starting 7 days after the induction of sAD; b. early stage, ES, starting 45 days after the induction of sAD; and c. late stage, LS, starting 90 days after the induction of sAD), the animal was always placed in the maze with its head toward the open arms. After each trial, the maze was cleaned with a solution of ethanol (70%) and left to dry (5 min) to avoid the influence of olfactory cues. The trained observer who was blinded to the division of animals into different groups analyzed behavioral responses.

### 2.5. Determination of Hematological and Immunological Parameters in the Blood and Spleen

In order to determine the level of peripheral inflammation, blood was collected two times: at the early (volume: 2–3 mL) and late (volume: 5 mL) stages of disease progression, via heart puncture, and at this time the animals were under inhalation anesthesia (2.5% isoflurane). The hematological parameters (number and percentage of various leukocyte populations, red blood and platelet cell system parameters) were determined in the heparinized whole blood with a hematology analyzer (Horriba, Loos, France).

Lymphocyte blood and spleen subpopulations were determined using cytometric analysis according to a method described previously [20,43,44], with some modification. In order to identify lymphocyte subpopulations, two types of three-color combinations of fluorescent monoclonal antibodies (powder form mixed with distilled water—0.5 mL/vial) were used: CD3:FITC/CD4:RPE/CD8:AlexaFluor 647 (Biorad, TC018, Hercules, CA, USA) and CD3:FITC/CD45RA:RPE/CD161:AlexaFluor 647 (Biorad, TC019, Hercules, CA, USA).

From the spleen, before cytometric assays, isolated spleen mononuclear cells (SMCs) were obtained. For this purpose, tissue of the spleen was placed in a solution of Hank’s balanced salt (HBSS Sigma-Aldrich Manufacturer Life Technologies, Paisley, UK) and HEPES/sodium bicarbonate ingredients, and the samples were then blended and centrifuged (30 min, 4 °C, 1113× *g*). The isolated SMCs were collected with a pasteur pipette, washed with a phosphate-buffered saline (PBS) suspension in RPMI-1640 with 10% calf bovine serum, and seeded at a 4 × 10^6^ cells/mL concentration and used in the flow cytometry analysis.

In a light-resistant test tube, 25 μL of whole-blood or isolated SMC samples were mixed with 25 μL of an antibody solution and incubated (30 min) in dark. Then, IO test 3 was added (25 µL, Beckman Coulter, A07800, Brea, CA, USA), and red blood cells were lysed by adding Versalyse solution (Beckman Coulter, A09777, Brea, CA, USA). In another step, 700 μL of PBS was added to the samples and they were mixed. The prepared solutions were protected from light and stored (4 °C) in darkness until flow cytometry had been performed with a Cytomics FC 500 flow cytometer (Beckman Coulter, Brea, CA, USA) and MXP Software version 2.1. The percentages of lymphocyte population/subpopulation were assessed during the analysis, displaying forward- and side-scatter characteristics. The total lymphocyte subpopulation numbers were obtained from the hematology counter (Horriba, Loos, France).

### 2.6. Detemination of Anti-Inflammatory Interleukin-10 (IL-10) and Pro-Inflammatory Interleukin-6 (IL-6) Concentrations in Plasma

In order to determine the level of peripheral inflammation, the concentrations of plasma cytokines, IL-10 and IL-6, were determined via the enzyme-linked immunoassay (ELISA) method by using commercially available Rat-IL-10 (sensitivity: 3 pg/mL) and Rat-IL-6 (sensitivity: 16 pg/mL) ELISA kits (catalog numbers: ERA23RB and ERA31RB, Invitrogen, Waltham, MA, USA). The absorbance was read using a DTX 880 Multimode Detector (Beckman Coulter, Brea, CA, USA) system set to 450 nm. Concentrations of IL-10 and IL-6 were calculated based on the standard curve generated by Beckman Coulter’s Biomek software version i5 program, based on the absorbance of standard samples.

### 2.7. Determination of Corticosterone (CORT) Concentration in Plasma

In order to determine the level of peripheral HPA axis activation, the corticosterone (CORT) concentrations were determined according to a method previously described [43]. Blood samples from rats were collected between 9:00 and 10.00 a.m. (the time of lowest concentration) through a cardiac puncture, and at this time animals were under inhalation anesthesia (2.5% isoflurane, airflow: 0.5 L/min). The CORT concentration in plasma was measured by a radioimmunoassay method using a commercially available kit (rCorticosterone (125I) RIA KIT, RK-548, isotop Institute of Isotopes Co., Ltd., Budapest, Hungary, sensitivity: 0.5 ng/mL) and Wizard 1470 gamma counter (Pharmacia—LKB, Turku, Finland). The determinations for each sample were made twice.

### 2.8. Sacrifice of Animal and Collection of Tissues and Organs

Animals were sacrificed under inhalation anesthesia (2.5% isoflurane, airflow: 0.5 L/min) by administering a lethal dose (2 mL/kg, i.p.) of Morbital (Biowet, Puławy, Poland). Blood was taken by cardiac puncture, and the thymus and spleen were collected. The spleen and thymus were weighed, and their relative weights were calculated according to their body weight (mg/100 g b.w.). After thymus and spleen collections, animals were transcardially perfused with PBS and 4% paraformaldehyde (PFA). Brains were post-fixed (overnight, 4% PFA) and cryoprotected (30% sucrose), and 30 μm coronal sections were cut (CM 1850, Leica Biosystems, Wetzlar, Germany). Coronal brain sections containing the placement of the guide cannulas were stained by the Nissl procedure and analyzed using Stemi 508 microscope and Zen 3.5—blue edition software (Zeiss, München, Germany). The representative photograph of microscopic slide showing Nissl-stained sections of the localization of the guide cannulas at the −1.3 mm Bregma level was shown in Figure S1.

### 2.9. Data Analysis

The data are presented as mean ± SD. For the power analysis, the sample size estimation was based on preliminary experiments and the previous literature, and sample size tools were available from Test power analysis, G*Power 3.1.9.7. Nonparametric tests (Statistica 13.1.3360 software, Statsoft, Tulsa, OK, USA) were performed because some parameters (Table S1) were characterized by non-homogeneous variance (Levene test) or the abnormal distribution of the variables (Kołmogorow–Smirnov test). Statistical analyses were performed using the Kruskal–Wallis ANOVA by rank for multiple comparisons (time: baseline conditions, a very early stage, an early stage, and a late stage; treatment: STZ, IGF-1, VEH, and SAL) and the Mann–Whitney U test for comparison between two groups of animals. Probability (*p*) values lower than 0.05 were regarded as being statistically significant.

Due to the varying blood volumes collected during the heart puncture and the occurrence of technical interference during recording in some cases, the statistical analysis was performed on a diverse range of data. The exact number of N for a single parameter is presented in the graphs.

## 3. Results

### 3.1. Behavioral Activity Associated with Anhedonia in the Sucrose Preference Test (SPT)

**Figure 2:**
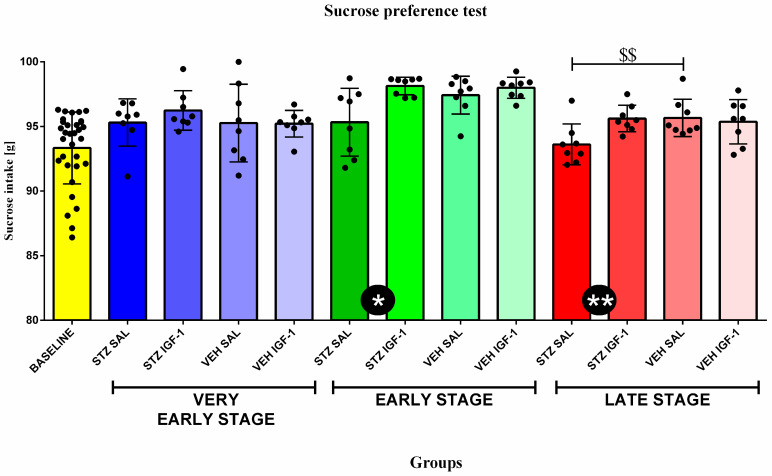
The effect of insulin-like growth factor-1 (IGF-1) treatment on behavioral activity associated with anhedonia in the sucrose preference test at the baseline conditions, at the very early, early, and late stages after intracerebroventricular injections of streptozotocin and saline (STZ SAL), streptozotocin and insulin-like growth factor-1 (STZ IGF-1), citrate buffer and saline (VEH SAL), and citrate buffer and insulin-like growth factor-1 (VEH IGF-1). Data are presented as the mean ± SD and were analyzed by using the Mann–Whitney U test. Explanations: * in a black circle—*p* < 0.05, ** in a black circle—*p* < 0.01, indicating the significance of differences between STZ SAL and STZ IGF-1; $$ above the lines—*p* < 0.01, indicating the significance of differences to VEH SAL.

Statistical analysis showed a significant difference (*p* < 0.05, *p* < 0.01, respectively) in sucrose consumption between STZ IGF-1 and STZ SAL animals, with STZ IGF-1 rats consuming more sucrose at the 4% sucrose dose at the early and late stages. Furthermore, we found that STZ SAL animals significantly (*p* < 0.01) decreased their intake of the 4% sucrose solution compared to VEH SAL rats at the late stage (Figure 2).

Table 1 shows the effect of time (stage of sAD) on sucrose intake. Compared to the baseline (before injection), rats from the STZ IGF-1 (*p* < 0.001), VEH SAL (*p* < 0.001), and VEH IGF-1 (*p* < 0.001) groups showed significantly increased sucrose solution intake measured at the early stage of sAD progression. In addition, at the very early stage, significantly higher (*p* < 0.05, *p* < 0.01, respectively) sucrose solution intake than at the baselines was observed in STZ SAL and STZ IGF-1 rats, and at the late phase significantly higher (*p* < 0.05) sucrose consumption than at the baseline was observed in the STZ IGF-1, VEH SAL, and VEH IGF-1 groups (in all comparisons, *p* < 0.05). Moreover, sucrose intake was lower at the late phase than at the early stage in the STZ IGF-1 (*p* < 0.01) and VEH IGF-1 (*p* < 0.01) groups, but sucrose consumption in these rats at the early stage was higher than at the very early phase (STZ IGF-1 *p* < 0.05, VEH IGF-1 *p* < 0.001).

### 3.2. Behavioral Activity Associated with Anxiety in the Elevated Plus Maze (EPM)

**Figure 3:**
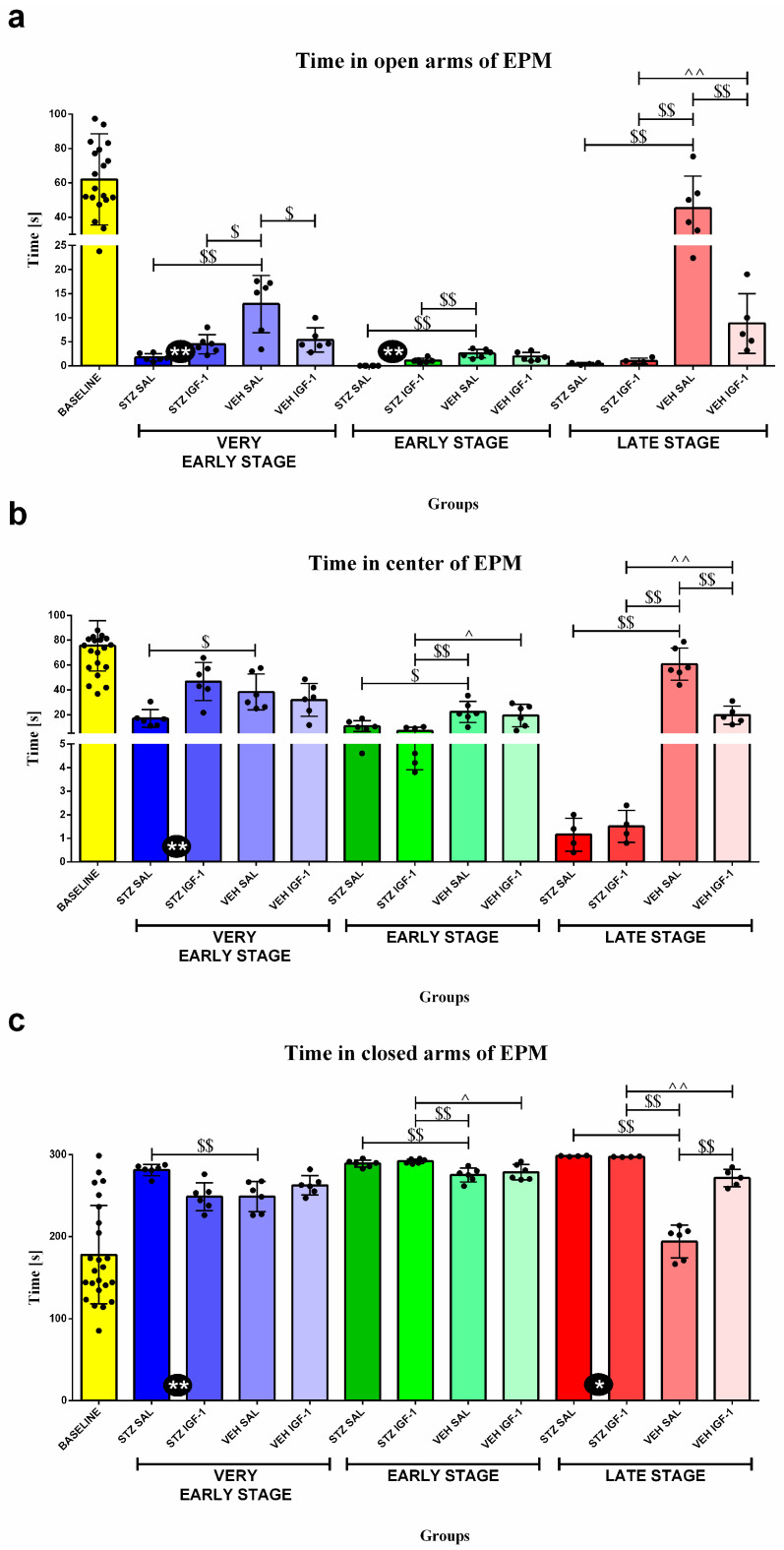
The effect of insulin-like growth factor-1 (IGF-1) treatment on behavioral activity associated with anxiety presented as the time spent in the open arms (**a**), the center (**b**), and the closed arms (**c**) of the elevated plus maze (EPM) at the baseline conditions, at the very early, early, and late stages after intracerebroventricular injections of streptozotocin and saline (STZ SAL), streptozotocin and insulin-like growth factor-1 (STZ IGF-1), citrate buffer and saline (VEH SAL), and citrate buffer and insulin-like growth factor-1 (VEH IGF-1). Data are presented as mean ± SD and were analyzed using a Mann–Whitney U test. Explanations: * in a black circle—*p* < 0.05, ** in a black circle—*p* < 0.01, indicating the significance of differences between STZ SAL and STZ IGF-1; $ above the lines—*p* < 0.05, $$ above the lines—*p* < 0.01, indicating the significance of differences to the VEH SAL group; ^ above the lines—*p* < 0.05, ^^ above the lines—*p* < 0.01, indicating the significance of differences between the STZ IGF-1 and VEH IGF-1 groups.

Figure 3 and Table 2 present behavioral activity assessed in the EPM test measured as time spent in the open arms, time spent in the center of the maze, time spent in the closed arms, at the baseline (before injection), and at the very early, early, and late stages of sAD progression. Rats from STZ IGF-1 spent more time than STZ SAL rats in the open arms of the maze at the very early and early stages (both *p* < 0.01) as well as in the center of the maze at the very early stage, and less time in closed arms at the very early (*p* < 0.01) and late stages (*p* < 0.05) of sAD.

Moreover, the time spent in the open arms of the VEH SAL group was longer compared to the (1) STZ SAL (*p* < 0.01), STZ IGF-1 (*p* < 0.05), and VEH IGF-1 (*p* < 0.05) groups at the very early stage; the (2) STZ SAL (*p* < 0.01) and STZ IGF-1 (*p* < 0.01) groups at the early stage; and the (3) STZ SAL (*p* < 0.01), STZ IGF-1 (*p* < 0.01), and VEH IGF-1 (*p* < 0.01) groups at the late stage of sAD progression. In addition, at the late stage, animals from the VEH IGF-1 group spent more time in the open arms than those from the STZ IGF-1 group (*p* < 0.01).

The time spent in the center of maze was longer for the VEH SAL group compared to (1) STZ SAL rats (*p* < 0.05) at the very early stage; (2) STZ SAL (*p* < 0.05), STZ IGF-1 (*p* < 0.01), and VEH IGF-1 (*p* < 0.05) rats at the early stage; and (3) STZ SAL, STZ IGF-1, and VEH IGF-1 animals (in all comparisons *p* < 0.01) at the late stage. Rats from STZ IGF-1 spent less time in the center than VEH IGF-1 rodents at the early (*p* < 0.05) and late stages (*p* < 0.01).

The time spent in the closed arms was shorter for VEH SAL rats compared to (1) STZ SAL rats at the very early stage (*p* < 0.01), the (2) STZ SAL and STZ IGF-1 groups (in both comparisons, *p* < 0.01) at the early stage, and (3) all the rats at the late stage (in all comparisons, *p* < 0.01). What is more, STZ IGF-1 animals spent more time in the closed arms than VEH IGF-1 rodents at the early (*p* < 0.05) and late stages (*p* < 0.01) of sAD.

Additionally, time spent in the open arms (1) at the very early stage in the STZ SAL, STZ IGF-1, VEH SAL, and VEH IGF-1 groups (in all comparisons, *p* < 0.001); (2) at the early stage in the STZ SAL, STZ IGF-1, VEH SAL, and VEH IGF-1 groups (in all comparisons, *p* < 0.001); and (3) at the late stage in STZ SAL (*p* < 0.01), STZ IGF-1 (*p* < 0.001), and VEH IGF-1 (*p* < 0.001) groups was shorter than at the baseline conditions. Time spent in the center was shorter (1) at the very early stage in the STZ SAL (*p* < 0.001), STZ IGF-1 (*p* < 0.01), VEH SAL (*p* < 0.001), and VEH IGF-1 (*p* < 0.001) groups; (2) at the early stage in the STZ SAL, STZ IGF-1, VEH SAL, and VEH IGF-1 groups (in all comparisons, *p* < 0.001); and (3) at the late stage in the STZ SAL (*p* < 0.01), STZ IGF-1 (*p* < 0.01), and VEH IGF-1 (*p* < 0.001) groups than at the baseline conditions. Time spent in the closed arms was longer (1) at the very early stage in the STZ SAL (*p* < 0.001), STZ IGF-1 (*p* < 0.05), VEH SAL (*p* < 0.05), and VEH IGF-1 (*p* < 0.01) groups; (2) the early stage in the STZ SAL (*p* < 0.001), STZ IGF-1 (*p* < 0.001), VEH SAL (*p* < 0.01), and VEH IGF-1 (*p* < 0.01) groups; and (3) the late stage in the STZ SAL (*p* < 0.01), STZ IGF-1 (*p* < 0.01), and VEH IGF-1 (*p* < 0.01) groups than at the baseline conditions. At the early stage all the rats spent less time in the open arms of the maze than at the very early stage (*p* < 0.01). In addition, rats from the STZ SAL group spent less time in the open arms at the late stage compared to the very early phase (*p* < 0.01), but more time compared to the early stage (*p* < 0.01). Moreover, STZ IGF-1 animals spent less time in the open arms (*p* < 0.01) at the late stage than the very early stage, and rats from the VEH SAL group spent more time in the open arms at the late stage than at the very early and early stages (in both comparisons, *p* < 0.01); the time spent in this part of the maze was longer in the VEH IGF-1 group at the late stage compared to the early stage (*p* < 0.01). In addition, time spent in the center was longer at the very early stage than the early and late phases of AD progression in the STZ SAL (*p* < 0.05, *p* < 0.01) and STZ IGF-1 groups (in both comparisons, *p* < 0.01). In the VEH SAL group, the time spent in the center at the very early phase was longer compared to the early stage (*p* < 0.05), but time at the very early stage was shorter than at the late stage (*p* < 0.05). At the late stage compared to the early phase, the time spent in the maze center was shorter (in both comparisons, *p* < 0.01) in the STZ SAL and STZ IGF-1 animals, but longer in the VEH SAL rats (*p* < 0.01). All of the rats spent more time in the closed arms at the early stage than at the very early stage (STZ SAL *p* < 0.05, STZ IGF-1 *p* < 0.01, VEH SAL *p* < 0.01, and VEH IGF-1 *p* < 0.05). Furthermore, the STZ SAL and STZ IGF-1 rats spent more time in the closed arms at the late than at the very early (STZ SAL, *p* < 0.05; STZ IGF-1, *p* < 0.01) and early (in both comparisons, *p* < 0.01) stages of sAD progression. The control VEH SAL animals spent more time in the closed arms at the very early and early stages than at the late phase of disease progression (in both comparisons, *p* < 0.01).

Moreover, Table S2 shows the effect of treatment and time on the number of entries to open arms, center, and closed arms, and the number of miction and defecations episodes in the EPM test.

### 3.3. Peripheral Blood Leukocytes and Their Subpopulations’ Numbers and Percentages

**Figure 4:**
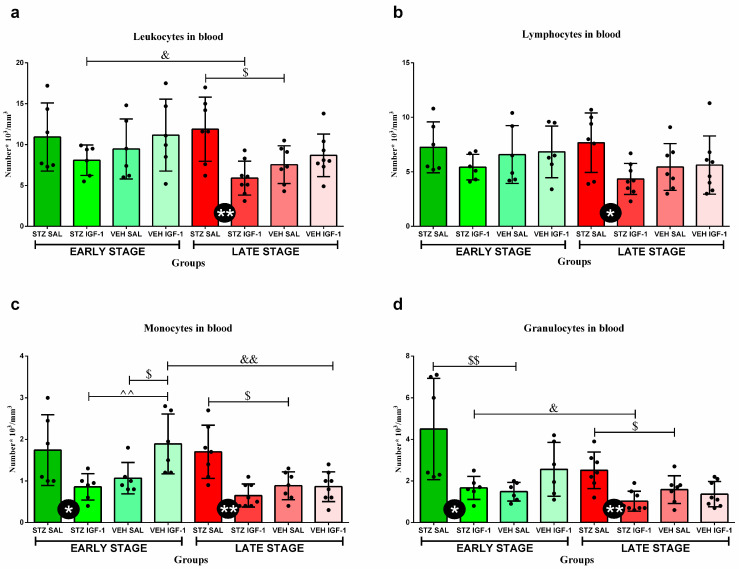
The effect of insulin-like growth factor-1 (IGF-1) treatment and time (stage) on the number of leukocytes (**a**), lymphocytes (**b**), monocytes (**c**), and granulocytes (**d**) at the early and late stages after intracerebroventricular injections of streptozotocin and saline (STZ SAL), streptozotocin and insulin-like growth factor-1 (STZ IGF-1), citrate buffer and saline (VEH SAL), and citrate buffer and insulin-like growth factor-1 (VEH IGF-1) in the peripheral blood. Data are presented as mean ± SD and were analyzed using the Mann–Whitney U test. Explanations: * in a black circle—*p* < 0.05, ** in a black circle—*p* < 0.01, indicating the significance of the differences between the STZ SAL and STZ IGF-1 groups; $ above the lines—*p* < 0.05, $$ above the lines—*p* < 0.01, indicating the significance of differences to the VEH SAL group; ^^ above the lines—*p* < 0.01, indicating the significance of differences between the STZ IGF-1 and VEH IGF-1 groups; and &—*p* < 0.05, &&—*p* < 0.01, indicating the significance of differences between the early and late stages of disease progression.

There were no significant differences in the total number of blood leukocytes and their populations, except for monocytes and granulocytes between the STZ SAL and STZ IGF-1 groups, with a lower cell number in STZ IGF-1 animals (*p* < 0.05) at the early stage of sAD progression (Figure 4). Moreover, at this stage, VEH IGF-1 rats had a higher number of monocytes than STZ IGF-1 (*p* < 0.01) and VEH SAL (*p* < 0.05) animals, and in the case of granulocytes STZ SAL rodents had a higher number of cells than the VEH SAL group (*p* < 0.01). However, at the late stage, a significantly lower total number of leukocytes (*p* < 0.01), lymphocytes (*p* < 0.05), monocytes (*p* < 0.01), and granulocytes (*p* < 0.01) in the blood was observed in STZ IGF-1 rats compared to STZ SAL animals. In addition, at this stage in STZ SAL rats, the number of leukocytes was higher than in the VEH SAL (*p* < 0.05) group. In the STZ IGF-1 group the number of WBCs was lower at the late stage than at the early stage (*p* < 0.05). What is more, the number of monocytes and granulocytes was higher in STZ SAL rats than in the VEH SAL group (in both comparisons, *p* < 0.05) at the late stage of sAD. Additionally, the number of monocytes in the VEH IGF-1 group was lower at the late phase than at the early stage (*p* < 0.01). The STZ IGF-1 group also had a lower number of granulocytes at this stage than at the early phase of sAD progression (*p* < 0.05).

The percentages of lymphocytes, monocytes, and granulocytes in the peripheral blood are shown in Table 3. VEH SAL rats had higher percentages of lymphocytes than VEH IGF-1 animals (*p* < 0.05) at the early phase of sAD. At this phase, animals from the STZ IGF-1 group had lower percentages of monocytes than VEH IGF-1 (*p* < 0.05) rats, but the percentages of granulocytes in VEH IGF-1 rodents were higher than in the VEH SAL (*p* < 0.05) group. The percentages of granulocytes at the late (*p* < 0.01) phases were lower in STZ IGF-1 animals than in the STZ SAL (*p* < 0.05) group. Moreover, in the STZ IGF-1 animals, the percentages of granulocytes at the late stage were lower than at the early stage of sAD (*p* < 0.05).

### 3.4. Peripheral Blood and Spleen Lymphocytes and Their Subpopulations’ Numbers and Percentages

**Figure 5:**
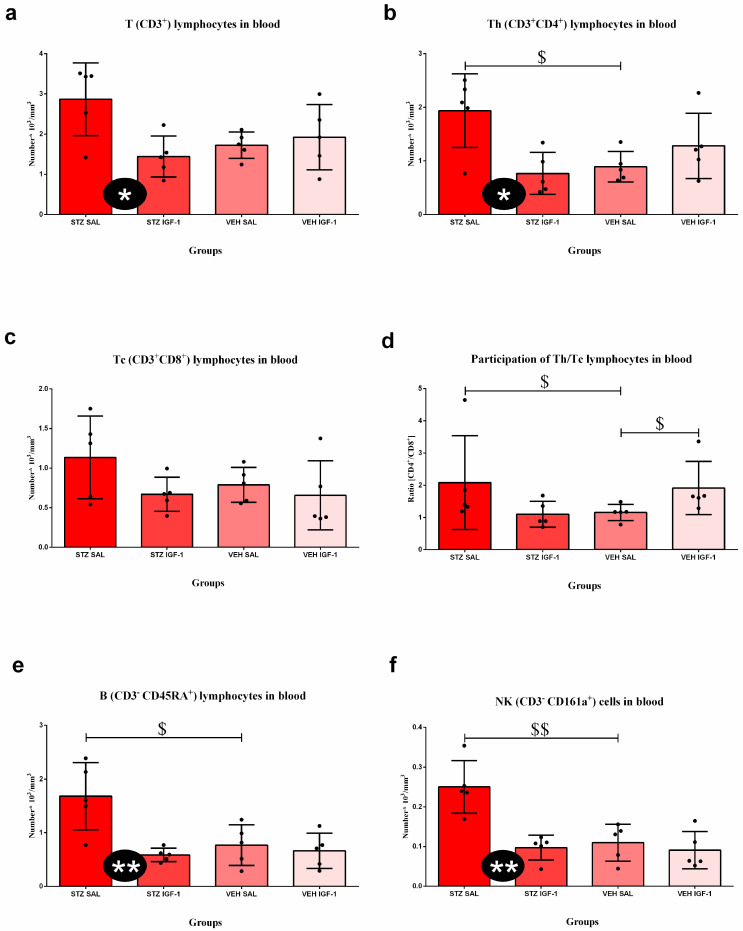
The effect of insulin-like growth factor-1 (IGF-1) treatment on the number of T CD3^+^ (**a**), Th CD4^+^ (**b**), Tc CD8^+^ (**c**), B CD45RA^+^ lymphocytes (**e**), NK cell CD161a^+^ (**f**), and ratio of CD4^+^/CD8^+^ lymphocytes (**d**) at the late stage after intracerebroventricular injections of streptozotocin and saline (STZ SAL), streptozotocin and insulin-like growth factor-1 (STZ IGF-1), citrate buffer and saline (VEH SAL), and citrate buffer and insulin-like growth factor-1 (VEH IGF-1) in the peripheral blood. Data are presented as mean ± SD and were analyzed using the Mann–Whitney U test. Explanations: * in a black circle—*p* < 0.05, ** in a black circle—*p* < 0.01, indicating the significance of differences between the STZ SAL and STZ IGF-1 groups; $ above the lines—*p* < 0.05, $$ above the lines—*p* < 0.01, indicating the significance of differences to the VEH SAL group.

At the late stage of sAD (Figure 5), the number of T CD3^+^, Th CD4^+^, B CD45RA^+^, and NK cell CD161a^+^ was lower in STZ IGF-1 animals than in the STZ SAL rodents (*p* < 0.05, *p* < 0.05, *p* < 0.01, and *p* < 0.01, respectively). Moreover, the VEH SAL group had a lower number of T helper lymphocytes than STZ SAL rats (*p* < 0.05), a lower ratio of Th/Tc cells than STZ SAL (*p* < 0.05) and VEH IGF-1 (*p* < 0.05) animals, and a lower number of B lymphocytes and NK cells than STZ SAL (*p* < 0.05, *p* < 0.01, respectively) group.

Table 4a shows the percentage of lymphocytes’ subpopulations in the blood. There were no significant differences in the percentages of T lymphocytes, B lymphocytes, and NK cells among groups. The percentage of Th lymphocytes was higher in the VEH IGF-1 group than in VEH SAL animals (*p* < 0.05). In addition, the percentage of Tc lymphocytes was higher in STZ IGF-1 rats than in STZ SAL rodents (*p* < 0.05).

Table 4b shows the percentages of T lymphocytes, B lymphocytes, and NK cells in the spleen. The percentage of T lymphocytes was higher in STZ IGF-1 animals than in STZ SAL (*p* < 0.05) and VEH SAL rats (*p* < 0.01). Moreover, the percentage of these cells was also higher in the VEH IGF-1 group than in the VEH SAL group (*p* < 0.01). The percentages of B lymphocytes and NK cells in STZ IGF-1 rodents were higher than in STZ SAL (in both comparisons, *p* < 0.05) and VEH SAL animals (in both comparisons, *p* < 0.01). In addition, the percentage of these cells was higher in the VEH IGF-1 group than in VEH SAL rats (in both comparisons, *p* < 0.05).

### 3.5. Relative Weights of the Spleen and Thymus

The relative weights of the spleen and thymus at the late phase of sAD progression are shown in Table 4c. There is no statistical difference in the relative weight of the spleen between the groups, but the relative weight of the thymus was higher in the STZ SAL group than in VEH SAL (*p* < 0.05) rats.

### 3.6. Hematological Parameters

The results of hematological parameters, along with their descriptions, are presented in Table S3.

### 3.7. The Concentrations of Interleukin 6 (IL-6) and Interleukin 10 (IL-10) in Plasma

**Figure 6:**
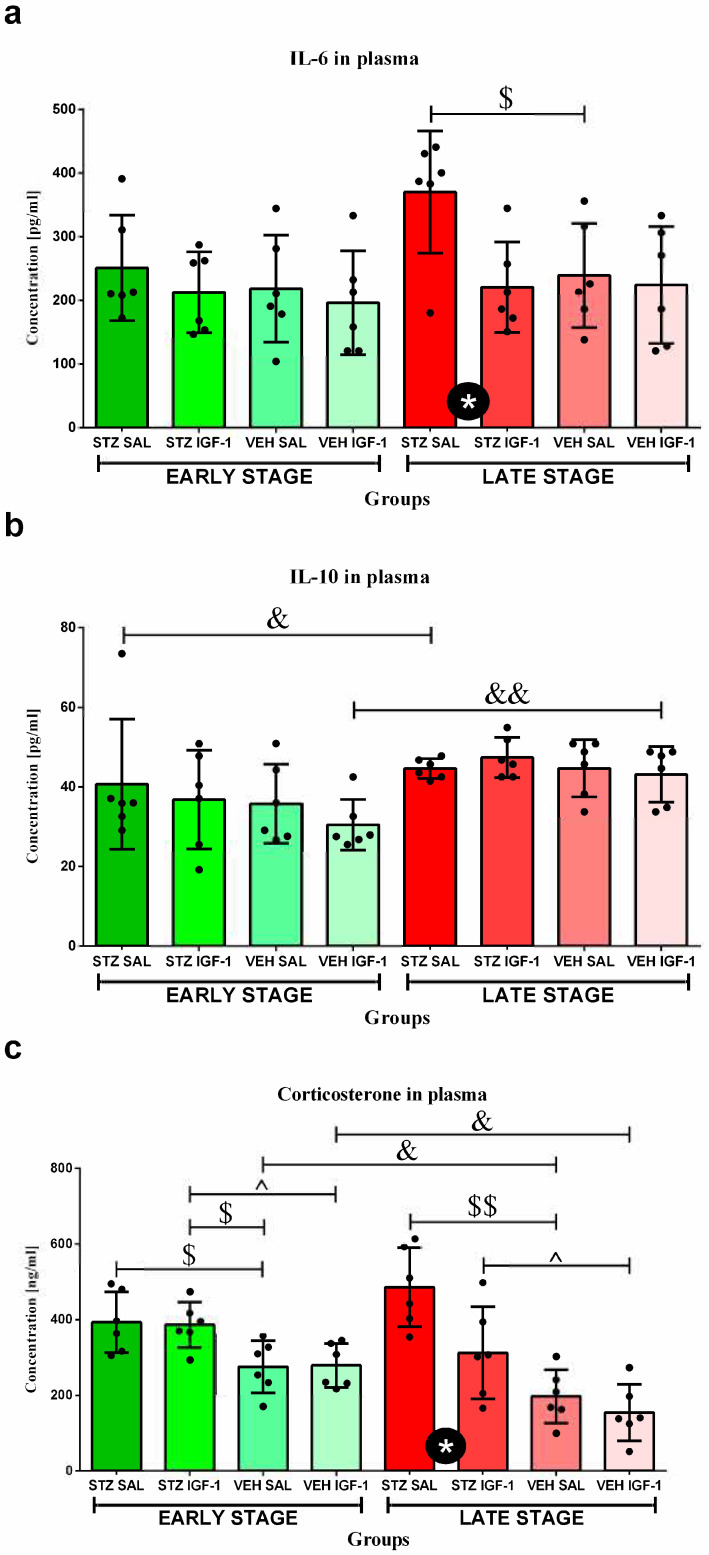
The effect of insulin-like growth factor-1 (IGF-1) treatment and time (stage) on the concentrations of interleukin-6 (IL-6) (**a**), interleukin-10 (IL-10) (**b**), and corticosterone (CORT) (**c**) at the early and late stages after intracerebroventricular injections of streptozotocin and saline (STZ SAL), streptozotocin and insulin-like growth factor-1 (STZ IGF-1), citrate buffer and saline (VEH SAL), and citrate buffer and insulin-like growth factor-1 (VEH IGF-1) in plasma. Data are presented as mean ± SD and were analyzed using the Mann–Whitney U test. Explanations: * in a black circle—*p* < 0.05, indicating, the significance of differences between STZ SAL and STZ IGF-1; $ above the lines—*p* < 0.05, $$ above the lines—*p* < 0.01, indicating the significance of differences to the VEH SAL group; ^ above the lines—*p* < 0.05, indicating the significance of differences between the STZ IGF-1 and VEH IGF-1 groups; and &—*p* < 0.05, &&—*p* < 0.01, indicating the significance of differences between the early and late stages of disease progression.

The concentration of IL-6 (Figure 6a) in the STZ SAL group was higher than in STZ IGF-1 and VEH SAL animals (in both comparisons, *p* < 0.05) at the late phase.

The concentration of IL-10 (Figure 6b) in the STZ SAL (*p* < 0.05) and VEH IGF-1 (*p* < 0.01) groups was higher at the late stage than at the early phase of sAD progression.

### 3.8. Determination of Plasma Corticosterone (CORT) Concentration

The concentration of corticosterone (CORT) in plasma (Figure 6c) was higher in STZ SAL and STZ IGF-1 rats than in the VEH SAL group (in both comparisons, *p* < 0.05) at the early stage. Moreover, at that phase, the CORT concentration was higher in STZ IGF-1 rats compared to animals from the VEH IGF-1 group (*p* < 0.05). At the late stage, STZ SAL rodents had higher concentrations of CORT than the STZ IGF-1 (*p* < 0.05) and VEH SAL (*p* < 0.01) groups. Moreover, the STZ IGF-1 group had higher CORT concentrations than VEH IGF-1 rats (*p* < 0.05). At that phase, the CORT concentrations were lower in VEH SAL and VEH IGF-1 rats than at the early phase of sAD progression (in both comparisons, *p* < 0.05).

## 4. Discussion

To the best of our knowledge, the results of our research demonstrate for the first time that ICVIGF-1 injections (total dose: 2 µg, administered twice, on days 1 and 3, 0.5 µg/ventricle/injection), simultaneously with the induction of the sporadic AD model by ICVSTZ injections (total dose: 3 mg/kg, administered twice, on days 2 and 4, 0.75 mg/kg/ventricle/injection), reduce the anhedonia and anxiety levels in the streptozotocin-induced sAD model. These positive effects were associated with a reduction in the peripheral inflammation state. Our data highlight that IGF-1 pretreatment in a STZ-induced model of sAD influences peripheral inflammation and corticosterone concentration, and can be a new therapeutic approach to treating anxiogenic/depressive behavior.

Neuropsychiatric symptoms, including apathy, depression, and anxiety, have been linked with cerebral Aβ deposition, neuronal loss, cognitive decline [45,46], and inflammation [47] in AD patients. The associations between anxiety and regional Aβ in the cingulate, prefrontal, and parietal cortices (which partly overlap with regions involved in Aβ accumulation in the very early stages of AD) were documented by Palmqvist et al. [48] and Johansson et al. [46]. Anhedonia and apathy levels are elevated in individuals with late-stage AD, and these symptoms have been associated with metabolic changes in the anterior cingulate cortex and other frontal regions [49,50], as well as the hippocampus, nucleus accumbens, thalamus, and putamen [46]. In our previous study, we showed that ICVIGF-1 administration in a rat STZ-induced model of AD reduced amyloid β (Aβ40–42) aggregation in the hippocampus. Our study proved a reduction in protein aggregation in the CA1 region of the hippocampus, prefrontal cortex, and nucleus accumbens (NAc) after IGF-1 premedication in a late STZ-induced rat model of AD [36]. In addition, a reduction in the number of CD68^+^ cells in the hippocampus was noted 90 days after ICVIGF-1 injection in a STZ-induced rat model of AD [36]. The CD68^+^ marker of cell activation is a lysosomal glycoprotein associated with increased phagocytic activity [51]. In a post mortem study of AD patients, CD68^+^ expression was strongly related to neuritic plaques and tangles, as well as the dementia score [52]. Taken together, the CD68^+^ marker of microglial phagocytic activation is present in brain tissue during the late phase of AD, when anhedonia and anxiety are observed [53,54]. Furthermore, our previous research [20] on an ICVSTZ sAD model showed that anxiety disorders, which were measured as less time spent in the open arms and the center of elevated plus maze (EPM) and more time spent in the closed arms of the maze, developed at different sAD stages. The progressive increase in anxiety levels induced by ICV-STZ administration in rats has also been reported by other groups [55,56], and such a change promotes the development of depressive behavior [57]. Therefore, in order to investigate the effect of ICVIGF-1 administration on depressive and anxiety behavior in the sAD model, we conducted two behavioral tests: the sucrose preference (SPT) test and EPM at three different stages of sAD progression. Our experiment showed that IGF-1 reduces anhedonia levels at the early (45 days after injection) and late (90 days after injection) stages of sAD in the SP test, as indicated by increased sucrose intake. Moreover, central IGF-1 administration decreased anxiety levels in the EPM, demonstrated as more time spent in the open arms and the center of the maze at 7 and 90 days after injection concomitantly with a higher number of entrances to the open arms of maze at the early stage of sAD. Other researchers have found similar results related to a reduction in depressive behavior mediated via increased IGF-1 acting at the IGF-1 receptor. Basta-Kaim et al. [35] showed that adult rats that were prenatally stressed exhibited depressive behavior in the forced-swim test, which was associated with reduced IGF-1 levels in the hippocampus and frontal cortex, while IGF-1 reversed this depressive disorder through influencing the IGF-1 receptor. Other authors demonstrated that IGF-1 led to the facilitation of fear extinction memory consolidation, pointing to IGF-1 as a key regulator of anxiety and its potential role as a novel therapeutic target for the treatment of anxiety [58]. In addition, other studies demonstrated that increasing the central level of IGF-1 by ICV injections of its binding protein inhibitor produced anxiolytic-like and antidepressant-like behavioral effects in the mouse in vivo, similar to IGF-1 administration [39].

The positive effects of ICVIGF-1 administration associated with the reduction in behavioral disorders related to anhedonia and anxiety observed in the present work are connected with decreased inflammation in the body. Our previous study showed that ICVIGF-1 injection reduced STZ-induced neuroinflammation, decreasing the number of microglia CD68^+^ cells and β-amyloid plaques in the hippocampus and thus improving spatial memory deficits [36]. The present results suggest that the improvements in spatial memory observed after ICVIGF-1 administration in the sAD model may also be attributed to IGF-1-induced reductions in anhedonia and anxiety and peripheral anti-inflammatory effects. Another study demonstrated that central injections of IGF-1 in a rat model of stroke had a protective effect on the brain–blood barrier (BBB), which prevented the development of neuroinflammation [59]. Current research has shown that IGF-1 also reduces peripheral inflammation, especially at the late stage of sAD progression. It was manifested by the reduced number of leukocytes, lymphocytes, T lymphocytes, Th lymphocytes, NK cells, B lymphocytes, monocytes, and granulocytes, as well as lower IL-6 concentrations in plasma. In AD subjects, IGF-1 signaling disorders were found [60], as in dwarf animals, in which IGF-1 administration decreased the total number of leukocytes and T lymphocytes [61]. In addition, we observed elevated corticosterone concentration at the early sAD stage, which decreased in the late phase of disease progression. This suggests the involvement of the HPA axis in reducing peripheral inflammation. In our opinion, increased levels of corticosterone, which has anti-inflammatory properties, early in the disease progression, combined with the administration of IGF-1, which also has anti-inflammatory properties, resulted in a decrease in the numbers of various leukocyte subpopulations late in the disease progression. We assume that this effect was only noticed at the late phase (90 days after ICVSTZ injection), because at the very early stage of the disease development (7 days after ICVSTZ injection) the corticosterone concentration was not high enough to induce an immunosuppressive effect. However, this hypothesis should at least be tested by measuring the corticosterone concentration at the very early phase. Two to four weeks after the peripheral administration of STZ in rats, an increased concentration of corticosterone was observed compared to healthy animals, yet its lowest concentration was recorded in week 3 in STZ-treated rodents [62]. This suggests that the concentration of this hormone is not high enough to trigger a sufficiently strong immunosuppressive reaction in the body during the first period of disease. On the other hand, we observed an increase in the percentage of T cytotoxic (CD8^+^) lymphocytes in the blood at the late stage of sAD. It is worth noting that IGF-1 plays a crucial role in thymopoiesis, the process of T cell development in the thymus [63]. When IGF-1 is administered in mice, it leads to an increase in the percentage of naive TCD4^+^ and TCD8^+^ lymphocytes in the periphery [64]. This suggests that IGF-1 enhances the thymic output of mature T cells. In addition, in the ICVIGF-1- and ICVSTZ-treated rats, the relative mass of the thymus was lower (statistically unconfirmed) than in rats with the sAD model, which may be an effect of T lymphocytes release to the peripheral blood. However, infiltrating TCD8^+^ lymphocytes triggered microglia activation and promoted behavioral deficits in the APP-PS1 transgenic mice [65]; therefore, the elevated level of TCD8^+^ lymphocytes in the peripheral blood may be the result of lymphocyte retention and improved BBB integrity. Some evidence in a rat model of ischemia supports this thesis. Bake et al. [66,67] showed that IGF-1 reduced the trafficking of immune cells to the ischemic site and promoted barrier function by increasing anchorage and stabilizing the cell geometry of surviving endothelial cells. In our study, we observed that IGF-1 decreased plasma IL-6 concentrations in our model of AD at the late stage of disease progression. In contrast, other studies, using a rat stroke model, demonstrated that 5 days after the model induction, ICVIGF-1 increased IL-6 plasma concentration, but the intraperitoneal injection of IGF-1 reduced this concentration [68]. This suggests that the anti-inflammatory effects of IGF-1, understood as a reduction in the concentration of pro-inflammatory cytokines, administered centrally occur via an additional mechanism (indirectly). It is obvious that this mechanism involves a reduction in the number of lymphocytes producing these cytokines.

Other authors observed an increase in the weight of the spleen and thymus, as well as the number of T, B, TCD4^+^, and TCD8^+^ cells in the spleen after IGF-1 administration [69]. Our research has shown that ICVIGF-1 injection increases the percentage of T and B lymphocytes, as well as NK cells in the spleen and also the relative spleen weight (statistically unconfirmed). This suggests that IGF-1 promotes the migration of immune cells from the periphery to the splenic pool, where they can be phagocytosed or await release in the event of the need for an increased immune response. However, the mechanism of IGF-1 effect on peripheral inflammation in AD requires further investigation, because this neurotrophin can strengthen the immune response even through the activation of the proliferation of T regulatory cells [70].

Moreover, the reduced activity of the HPA axis observed at the late stage of sAD may be the cause of the reduction in depressive-like behavior at this stage. Although the direct effects of IGF-1 on the HPA axis are currently unknown, it is worth noting that corticosterone and IGF-1 can carry out their activities through the same pathway. Studies on the model of chronic unpredictable stress have shown that IGF-1 exerts its anti-inflammatory effect by activating the PI3K/Akt/FoxO3 pathway [71], and a component of this pathway, PI3K/Akt, is responsible for antidepressant effects [72]. Corticosterone inhibits this pathway, and thus induces the cell death of hippocampal neurons, while IGF-1 administration eliminates this effect. This suggests that restoring normal HPA axis activity and, consequently, reduced plasma corticosterone levels are neuroprotective in an Alzheimer’s disease model.

A primary limitation of our study is the absence of direct assessments verifying that the ICVIGF-I pretreatment modulates brain glucose metabolism and counteracts the effects induced by STZ administration. Nonetheless, multiple previous studies describing the properties of IGF-1 (for a review, see [73]) support the assumption that a similar mechanism may have been active under our experimental conditions. Furthermore, the impact of sex differences on the prevalence of sAD and differences in the immune response between female and male rats, as well as behavior and inflammatory outcomes over a longer period of time, e.g., 6 months after ICV injections of IGF-1 and STZ, should be investigated in future studies. However, our current results clearly indicate positive effects of this neurotrophin on a sAD model and provide a solid basis for the development of effective treatments of depression and anxiety associated with AD in humans.

## 5. Conclusions

Our results show that ICV injections of IGF-1 (total dose: 2 µg) reduce the anxiety and anhedonia disturbances in an ICVSTZ model of sporadic Alzheimer’s disease in rats. IGF-1 induced beneficial behavioral changes by reducing peripheral inflammation (a decreased number of leukocytes, lymphocytes, T lymphocytes, Th lymphocytes, monocytes, and granulocytes in the peripheral blood; reduced IL-6 concentration in plasma) and reduced the HPA axis response (reduced corticosterone concentration in plasma) at the late stage (90 days after ICVSTZ administration) of sAD progression. This suggests that IGF-1, through its anti-inflammatory, anti-anxiety, and antidepressant properties, may be a novel therapeutic agent for the treatment of neuropsychiatric disorders in sAD. However, further research is necessary that takes into consideration additional variables, such as the longer development of the disease or the sex of the individuals.

## Figures and Tables

**Table 1:** The effect of time (stage) on behavioral activity associated with anhedonia in the sucrose preference test at baseline conditions, at the very early, early, and late stages after intracerebroventricular injections of streptozotocin and saline (STZ SAL), streptozotocin and insulin-like growth factor-1 (STZ IGF-1), citrate buffer and saline (VEH SAL), and citrate buffer and insulin-like growth factor-1 (VEH IGF-1).

Group	Phase	Sucrose Intake (g)
BASELINE	Baseline	93.34 ± 2.78
STZ SAL	Very early stage	95.31 ± 1.82 ^@^
STZ IGF-1		96.24 ± 1.53 ^@@^
VEH SAL		95.26 ± 3.01
VEH IGF-1		95.21 ± 1.03
STZ SAL	Early stage	95.33 ± 2.62
STZ IGF-1		98.13 ± 0.68 ^@@@%^
VEH SAL		97.42 ± 1.47 ^@@@^
VEH IGF-1		98.00 ± 0.82 ^@@@%%%^
STZ SAL	Late stage	93.61 ± 1.59
STZ IGF-1		95.62 ± 1.03 ^@&&^
VEH SAL		95.67 ± 1.45 ^@^
VEH IGF-1		95.36 ± 1.71 ^@&&^

Explanations: Data are presented as mean ± SD and were analyzed by using the Mann–Whitney U test. @—*p* < 0.05, @@—*p* < 0.01, and @@@—*p* < 0.001, indicating the significance of differences to baseline conditions; %—*p* < 0.05 and %%%—*p* < 0.001, indicating the significance of differences between very early and early stage/late stage; and &&—*p* < 0.01, indicating the significance of differences between early and late stages of disease progression.

**Table 2:** The effect of time (stage) on behavioral activity associated with anxiety presented as the time spent in the open arms (**a**), the center (**b**), and the closed arms (**c**) of the elevated plus maze (EPM) at the baseline conditions, at the very early, early, and late stages after intracerebroventricular injections of streptozotocin and saline (STZ SAL), streptozotocin and insulin-like growth factor-1 (STZ IGF-1), citrate buffer and saline (VEH SAL), and citrate buffer and insulin-like growth factor-1 (VEH IGF-1).

Group	Phase	Time (s)		
		a. Open Arms	b. Center	c. Closed Arms
BASELINE	Baseline	62.04 ± 26.48	75.55 ± 20.26	178.03 ± 60
STZ SAL	Very early stage	1.77 ± 0.77 ^@@@^	16.93 ± 7.17 ^@@@^	281.3 ± 7.04 ^@@@^
STZ IGF-1		4.47 ± 2 ^@@@^	46.77 ± 15.37 ^@@^	248.77 ± 17.03 ^@^
VEH SAL		12.83 ± 5.95 ^@@@^	38.30 ± 14.48 ^@@@^	248.87 ± 18.44 ^@^
VEH IGF-1		5.37 ± 2.52 ^@@@^	31.93 ± 13.19 ^@@@^	262.70 ± 11.77 ^@@^
STZ SAL	Early stage	0 ± 0 ^@@@%%^	10.77 ± 4.36 ^@@@%^	289.23 ± 4.36 ^@@@%^
STZ IGF-1		1.07 ± 0.53 ^@@@%%^	6.9 ± 3 ^@@@%%^	292.03 ± 2.7 ^@@@%%^
VEH SAL		2.53 ± 0.88 ^@@@%%^	22.17 ± 8.58 ^@@@%^	275.3 ± 8.51 ^@@%%^
VEH IGF-1		1.9 ± 0.9 ^@@@%%^	19.33 ± 8.95 ^@@@^	278.77 ± 9.49 ^@@%^
STZ SAL	Late stage	0.45 ± 0.19 ^@@%%&&^	1.15 ± 0.7 ^@@%%&&^	299 ± 0.71 ^@@%&&^
STZ IGF-1		1 ± 0.59 ^@@@%%^	1.5 ± 0.68 ^@@%%&&^	298.5 ± 1.11 ^@@%%&&^
VEH SAL		45.27 ± 18.77 ^%%&&^	60.70 ± 12.98 ^%&&^	194.03 ± 19.98 ^%%&&^
VEH IGF-1		8.8 ± 6.21 ^@@@%%^	19.68 ± 7.35 ^@@@^	271.52 ± 10.52 ^@@^

Explanations: Data are presented as mean ± SD and were analyzed using the Mann–Whitney U test. @—*p* < 0.05, @@—*p* < 0.01, and @@@—*p* < 0.001, indicating the significance of differences to the baseline conditions; %—*p* < 0.05, %%—*p* < 0.01, indicating the significance of differences between the very early and early stage/late stage; and &&—*p* < 0.01, indicating the significance of differences between the early and late stages of disease progression.

**Table 3:** The effect of insulin-like growth factor-1 (IGF-1) treatment and time (stage) on the percentage of lymphocytes, monocytes, and granulocytes at the early (**a**) and late stages (**b**) after intracerebroventricular injections of streptozotocin and saline (STZ SAL), streptozotocin and insulin-like growth factor-1 (STZ IGF-1), citrate buffer and saline (VEH SAL), and citrate buffer and insulin-like growth factor-1 (VEH IGF-1) in the peripheral blood.

	Group	Phase	Percentage of Lymphocytes [%]	Percentage of Monocytes [%]	Percentage of Granulocytes [%]
a.	STZ SAL	EARLY STAGE	67.93 ± 5.09	14.03 ± 3.43	18.05 ± 1.86
	STZ IGF-1		68.28 ± 7.53	10.78 ± 2.14 ^^^	20.94 ± 6.73
	VEH SAL		73.17 ± 6.99	11.37 ± 2.33	15.47 ± 5.75
	VEH IGF-1		62.45 ± 7.96 ^$^	14.03 ± 2.51	23.53 ± 6.52 ^$^
b.	STZ SAL	LATE STAGE	67.64 ± 7.51	13.89 ± 3.20	21.47 ± 6.30
	STZ IGF-1		74.38 ± 9.41	10.76 ± 2.79	13.61 ± 5.19 *^&^
	VEH SAL		73.17 ± 6.99	11.37 ± 2.33	15.47 ± 5.75
	VEH IGF-1		62.45 ± 7.96	14.03 ± 2.51	23.53 ± 6.52

Explanations: * *p* < 0.05 indicates significance of differences between STZ SAL and STZ IGF-1, $—*p* < 0.05 indicates significance of differences to VEH SAL, ^—*p* < 0.05 indicates significance of differences between STZ IGF-1 and VEH IGF-1, &—< 0.05 indicates significance of differences between early and late stage of disease progression.

**Table 4:** The effect of insulin-like growth factor-1 (IGF-1) treatment on the percentages of T CD3^+^, Th CD4^+^, Tc CD8^+^, B CD45RA^+^ lymphocytes, and NK cell CD161a^+^ in the blood (**a**); T CD3^+^, B CD45RA^+^ lymphocytes, and NK cell CD161a^+^ in the spleen (**b**). The relative weight of the spleen and thymus (**c**) at the late stage after intracerebroventricular injections of streptozotocin and saline (STZ SAL), streptozotocin and insulin-like growth factor-1 (STZ IGF-1), citrate buffer and saline (VEH SAL), and citrate buffer and insulin-like growth factor-1 (VEH IGF-1).

a.	**Group**	**Percentage of Blood T (CD3^+^) Lymphocytes [%]**	**Percentage of Blood Th (CD3^+^CD4^+^) Lymphocytes [%]**	**Percentage of Blood Tc (CD3^+^CD8^+^) Lymphocytes [%]**	**Percentage of Blood B (CD45RA^+^)** **Lymphocytes [%]**	**Percentage of Blood NK** **(CD161a^+^) Cells [%]**
	STZ SAL	35.81 ± 5.97	23.88 ± 5.58	14.12 ± 4.42	20.47 ± 1.61	3.48 ± 1.67
	STZ IGF-1	41.19 ± 8.35	21.99 ± 9.12	19.15 ± 3.05 *	17.35 ± 3.76	2.96 ± 1.28
	VEH SAL	37.60 ± 4.01	17.28 ± 3.38	16.95 ± 5.83	13.80 ± 5.89	2.17 ± 0.94
	VEH IGF-1	34.28 ± 7.27	28.74 ± 9.51 ^$^	13.84 ± 5.05	14.66 ± 5.32	2.11 ± 0.95
b.	**Group**	**Percentage of Spleen T (CD3^+^) Lymphocytes [%]**	**Percentage of Spleen B (CD45RA^+^)** **Lymphocytes [%]**		**Percentage of Spleen NK** **(CD161a^+^) cells [%]**	
	STZ SAL	22.70 ± 10.76	8.96 ± 3.88		2.29 ± 1.06	
	STZ IGF-1	43.59 ± 10.55 *^$$^	18.58 ± 3.88 *^$$^		4.91 ± 1.51 *^$$^	
	VEH SAL	16.82 ± 2.97	11.12 ± 1.39		1.42 ± 0.64	
	VEH IGF-1	39.34 ± 15.33 ^$$^	14.54 ± 2.66 ^$^		3.64 ± 1.66 ^$^	
c.	**Group**	**Relative Weight of Spleen (mg/kg b.w.)**		**Relative Weight of Thymus (mg/kg b.w.)**		
	STZ SAL	178.07 ± 23.40		55.69 ± 12.42 ^$^		
	STZ IGF-1	187.56 ± 25.46		52.56 ± 14.91		
	VEH SAL	175.35 ± 20.16		43.15 ± 10.58		
	VEH IGF-1	180.60 ± 9.93		44.97 ± 12.97		

Explanations: *—*p* < 0.05, indicating the significance of differences between the STZ SAL and STZ IGF-1 groups; $—*p* < 0.05, $$—*p* < 0.01, indicating the significance of differences to the VEH SAL group.

## Data Availability

Data are contained within the article and Supplementary Materials.

## Supplementary Materials

The following supporting information can be downloaded at: https://www.mdpi.com/article/10.3390/cells14151189/s1, Figure S1. Photograph of microscopic slide showing a Nissl-stained section of a brain of a representative rat. The asterisk indicates the area where the injection needle was inserted into the lateral ventricle. Images were obtained using a Stemi 508 Zeiss microscope equipped with imaging software (Zen 3.5—blue edition) at a magnification of 0.5 × 0.65. Table S1. The results obtained from the Levene test. Table S2. The effect of insulin-like growth factor-1 (IGF-1) treatment and time (stage) on the behavioral activity associated with anxiety, presented as the entrances to the open arms, center, and closed arms, and the number of miction and defecation in the elevated plus maze (EPM) at the baseline conditions, at the very early, early, and late stages after intracerebroventricular injections of streptozotocin and saline (STZ SAL), streptozotocin and insulin-like growth factor-1 (STZ IGF-1), citrate buffer and saline (VEH SAL), and citrate buffer and insulin-like growth factor-1 (VEH IGF-1). Table S3. The effect of insulin-like growth factor-1 (IGF-1) treatment and time (stage) on the number of red blood cells (RBCs), hemoglobin concentration (HGB), mean hemoglobin concentration in the red blood cells (MCHC), mean mass of the hemoglobin in the red blood cells (MCH), mean corpuscular volume (MCV), hematocrit (HCT), and red cell distribution width (RDW) (**a**); the number of platelets (PLTs), mean platelet volume (MPV), and platelecrit (PCT) (**b**) at the early and late stages after intracerebroventricular injections of streptozotocin and saline (STZ SAL), streptozotocin and insulin-like growth factor-1 (STZ IGF-1), citrate buffer and saline (VEH SAL), and citrate buffer and insulin-like growth factor-1 (VEH IGF-1) in the peripheral blood.

## Abbreviations

The following abbreviations are used in this manuscript:

IGF-1	Insulin-like growth factor-1
AD	Alzheimer’s disease
sAD	Sporadic Alzheimer’s disease
STZ	Streptozotocin
VEH	Vehicle
SAL	Saline
ICV	Intracerebroventricular injection
VES	Very early state of sAD progression
ES	Early state of sAD progression
LS	Late state of sAD progression
IL-6	Interleukin 6
IL-10	Interleukin 10
IL-17	Interleukin 17
EPM	Elevated plus maze
SPT	Sucrose preference test
GC	Glicocorticoid
BBB	Brain–blood barrier
ROS	Reactive oxygen species
CORT	Corticosterone
Th	T helper lymphocytes
Tc	T cytotoxic lymphocytes
Aβ	Beta amyloid
IRBS	Insulin-resistant brain state
HPA axis	Hypothalamic–pituitary–adrenal axis
PBS	Phosphate-buffered saline
RBC	Red blood cell
HGB	Hemoglobin concentration
MCHC	Mean hemoglobin concentration in the red blood cells
MCH	Mean mass of the hemoglobin in the red blood cells
MCV	Mean corpuscular volume
HCT	Hematocrit
RDW	Red cell distribution width
PLT	Platelet
MPV	Mean platelet volume
PCT	Platelecrit
