# Supplementary material for: Central Insulin-Like Growth Factor-1-Induced Anxiolytic and Antidepressant Effects in a Rat Model of Sporadic Alzheimer’s Disease Are Associated with the Peripheral Suppression of Inflammation
Source: Cells. 2025 Aug 1;14(15):1189. doi: 10.3390/cells14151189 (PMC12346486; doi:10.3390/cells14151189)
Supplement: Supplementary file 1 [file cells-14-01189-s001.zip › cells-3766828-supplementary/Table S1.pdf]

Table S1.The results obtained from Levene test.

Parameters	p
Consumption of sucrose (SPT)	0.00231
Time in the center (EPM)	0.000918
Time in the open arms (EPM)	0.0000001
Time in the closed arms (EPM)	0.0000001
Entrances to the center (EPM)	0.0006
Entrances to the open arms (EPM)	0.000006
Entrances to the closed arms (EPM)	0.09
Miction (EPM)	0.000764
Defecation (EPM)	0.001
Number of leukocytes in the blood	0.19
Number of lymphocytes in the blood	0.52
Number of monocytes in the blood	0.004509
Number of granulocytes in the blood	0.0000001
Percentage of lymphocytes in the blood	0.10
Percentage of monocytes in the blood	0.43
Percentage of granulocytes in the blood	0.015673
Number of red blood cells in the blood	0.80
Hemoglobin concentration	0.43
Hematocrit	0.35
Mean corpuscular volume	0.07
Mean mass of the hemoglobin	0.012161
Mean hemoglobin concentration	0.041055
Red cell distribution width	0.336676
Number of platelets in the blood	0.000015
Mean platelet volume	0.11
Platelecrit	0.0000001
Number of T lymphocytes in the blood	0.19
Number of Th lymphocytes in the blood	0.62
Number of Tc lymphocytes in the blood	0.06
Number of B lymphocytes in the blood	0.10
Number of NK cells in the blood	0.68

Ratio Th/Tc lymphocytes in the blood	0.08
Percentage of T lymphocytes in the blood	0.66
Percentage of Th lymphocytes in the blood	0.008132
Percentage of Tc lymphocytes in the blood	0.54
Percentage of B lymphocytes in the blood	0.026031
Percentage of NK cells in the blood	0.72
Percentage of T lymphocytes in the spleen	0.23
Percentage of B lymphocytes in the spleen	0.42
Percentage of NK cells in the spleen	0.38
IL-6 concentration in the plasma	0.99
IL-10 concentration in the plasma	0.11
Corticosterone concentration in the plasma	0.50
Relative weights of spleen	0.34
Relative weights of thymus	0.89
