# Supplementary material for: Central Insulin-Like Growth Factor-1-Induced Anxiolytic and Antidepressant Effects in a Rat Model of Sporadic Alzheimer’s Disease Are Associated with the Peripheral Suppression of Inflammation
Source: Cells. 2025 Aug 1;14(15):1189. doi: 10.3390/cells14151189 (PMC12346486; doi:10.3390/cells14151189)
Supplement: Supplementary file 1 [file cells-14-01189-s001.zip › cells-3766828-supplementary/Table S2.pdf]

Table S2. The effect of insulin-like growth factor-1 (IGF-1) treatment and time (stage) on behavioral activity associated with anxiety presented as the entrances to the open arms, center and closed arms and number of miction and defecation in elevated plus maze (EPM) at baseline conditions, at the very early, early, and late stage after intracerebroventricular injections of: streptozotocin and saline (STZ SAL), streptozotocin and insulin-like growth factor-1 (STZ IGF-1), citrate buffer and saline (VEH SAL), citrate buffer and insulin-like growth factor-1 (VEH IGF-1).

**Groups**	**Phase**	**Enters (No.)**			**Episodes (No.)**	
		Open arms	Center	Close arms	Miction	Defecation
Baseline	Baseline	9.67±4.22	21.04±6.99	12±4.88	4±1.93	0±0
STZ SAL	VERY EARLY STAGE	1.67±0.52[$@@@]	4.5±1.25[$@@@]	3.67±1.4[@@@]	1.83±0.41[@@]	0±0
STZ IGF-1		2.83±0.97[@@@]	10.2±4.66[@@]	8±4	1.17±0.41[*@@]	0±0
VEH SAL		3.6±1.82[@@]	10.5±4.72[@@]	7.2±3.42[@]	1.33±0.52[@@]	0±0
VEH IGF-1		3.4±0.89[@@]	10.33±2.86[@@]	8.2±2.05[@]	1.67±0.82[@@]	0±0
STZ SAL	EARLY STAGE	0±0[$$%%@@@]	3.33±1.63[$@@@]	3.33±1.63[$@@@]	2±0.89[@]	1.67±0.82[$$@@@%%]
STZ IGF-1		1.33±0.82[**@@@]	3.67±1.97[$%@@@]	3.33±1.37[$%@@@]	1.33±0.52[@@]	0±0[**]
VEH SAL		1.67±0.61[%@@@]	7±3.35[@@@]	6.83±2.93[@@]	1.67±0.52[@@]	0±0
VEH IGF-1		1.5±0.55[%%@@@]	6.17±2.32[%@@@]	5.5±2.66[$@@]	1.17±0.41[@@]	0±0

STZ SAL	LATE STAGE	$1\pm0^{\&\&@@}$	$1\pm0^{\$\%\%\&@@}$	$1\pm0^{\$\$\&@@}$	$0\pm0^{\$\$@@\%\%\&\&}$	$0\pm0^{\&\&}$
STZ IGF-1		$1.25\pm0.5^{\wedge @@}$	$2.25\pm1.26^{\wedge\%@@}$	$2\pm0.71^{\$\wedge\%@@}$	$1\pm0^{*\wedge\wedge @@}$	0±0
VEH SAL		$5.33\pm1.99^{@}$	$9.67\pm4.89^{@@@}$	$5.83\pm2.31^{@@}$	$1.33\pm0.52^{@@}$	0±0
VEH IGF-1		$3.6\pm1.14^{\&\&@@}$	$6.4\pm2.51^{@@@}$	$4.8\pm1.30^{\%@@}$	$2.6\pm0.89^{\$\&\&}$	0±0

Explanations: *-p<0.05, **-p<0.01 indicate significance of differences between STZ SAL and STZ IGF-1, ^-p<0.05, ^^-p<0.01 indicate significance of differences between STZ IGF-1 and VEH IGF-1, %-p<0.05, %%-p<0.01 indicate significance of differences between very early and early stage/late stage, $-p<0.05, $$-p<0.01 indicate significance of differences to VEH SAL, &-p<0.05, &&-<0.01 indicate significance of differences between early and late stage of disease progression, @-p<0.05, @@-p<0.01, @@@-p<0.001 indicate significance of differences to baseline conditions.

Results description: In all the groups (STZ SAL, STZ IGF-1, VEH SAL, VEH IGF-1) rats more frequently entered the open arms at baseline conditions than at all the stages (very early stage: p<0.001, p<0.001, p<0.01 p<0.01; early stage: in all comparisons p<0.001; late stage: p<0.01, p<0.01, p<0.05 p<0.01 respectively). At the very early stage, VEH SAL rats entered the open arms more often than STZ SAL rats (p<0.05). At the early stage, STZ SAL rodents entered these arms less frequently than VEH SAL (p<0.01) and STZ IGF-1 groups (p<0.01). In addition, at the late stage, STZ IGF-1 group also less frequently visited the open arms than VEH IGF-1 (p<0.05) animals. STZ SAL rats less frequently en-tered the open arms at the early stage than at the very early stage (p<0.01) and late stage (p<0.01). Rats from VEH SAL group also less frequently visited this part of maze at the early stage compared to the very early stage (p<0.05). Moreover, VEH IGF-1 rats entered open arms less frequently at the early stage than at the very early and late stages (in both comparisons p<0.01).

In all the groups (STZ SAL, STZ IGF-1, VEH SAL, VEH IGF-1) rats more frequently visited the center of the maze at baseline conditions than at all the stages of sAD progression (very early stage: p<0.001, p<0.01, p<0.01 p<0.01; early stage: all comparisons p<0.001; late stage: p<0.01, p<0.01, p<0.001 p<0.001 respectively). The center of the maze was more frequently visited by VEH SAL rats compared to STZ SAL group at the very early, early and late stages (in all comparisons p<0.05). Moreover, rats from STZ IGF-1 group less frequently entered the maze center than (1) VEH SAL rats at the early stage (p<0.05) and (2) VEH IGF-1 animals at the late stage (p<0.05). STZ IGF-1and VEH IGF-1 groups entered less frequently the maze center at the early stage than at the very early stage (in both comparisons p<0.05). STZ SAL rats entered the center less often at the late stage than at the very early and early stages (p<0.01, p<0.05), and STZ IGF-1 also less frequently visited this part of the maze at the late stage than at the very early stage (p<0.05).

The closed arms of maze at baseline conditions was visit more frequently than at the very early stage in STZ SAL (p<0.001), VEH SAL (p<0.05), VEH IGF-1 (p<0.05) groups, at the early (p<0.001, p<0.001, p<0.01 p<0.01 respectively) and late stage (all comparisons p<0.01) in all

the groups (STZ SAL, STZ IGF-1, VEH SAL, VEH IGF-1). Moreover at the early stage VEH SAL animals often enters to closed arms than STZ SAL, STZ IGF-1 and VEH IGF-1 animals (all comparisons $p<0.05$). In addition also VEH SAL rodent more frequently visit closed arms at the late stage than STZ SAL ($p<0.01$), and STZ IGF-1 ($p<0.05$) animals but STZ IGF-1 at this time rarely visit this part of maze than VEH IGF-1 rodents ($p<0.05$). At the very early stage, STZ IGF-1 animals visit closed arms more frequently than at the early stage ($p<0.05$) and late stage ($p<0.05$). In addition STZ SAL rodent more frequently visit closed arms at the early than late stage ($p<0.05$). Additionally at the very early stage VEH IGF-1 group more frequently enter closed arms than at the late stage ($p<0.05$).

In all the groups (STZ SAL, STZ IGF-1, VEH SAL, VEH IGF-1) the number of miction instances was higher at baseline conditions than at the very early (in all comparisons $p<0.01$) and the early stage ($p<0.05$, $p<0.01$, $p<0.01$, $p<0.01$ respectively). In addition also the number of miction episodes in STZ SAL, STZ IGF-1, and VEH SAL groups was higher at the late stage compared to baseline conditions (in all comparisons $p<0.01$). In STZ IGF-1 group, the number of miction instances at the very early stage was lower but at the late stage higher than in STZ SAL (in both comparisons $p<0.05$) rats. What's more, at the late stage, the number of miction episodes in STZ SAL animals was lower than in VEH SAL ($p<0.01$) animals and compared to the very early and early stages (in both comparisons $p<0.01$). In addition, VEH IGF-1 rats had a higher number of miction instances compared to VEH SAL ($p<0.05$) and STZ IGF-1 ($p<0.01$) at the late stage. Compared to baseline conditions, at the early stage of sAD progression the higher number of defecation in STZ SAL rodents was observed ($p<0.001$). Moreover, at the early stage, the number of defecation episodes in STZ SAL group was higher than in STZ IGF-1 and VEH SAL rats ($p<0.01$). In addition, in STZ SAL group at early phase number of defecation was higher than at very early and late stages (in all comparisons $p<0.01$).
