# Supplementary material for: Central Insulin-Like Growth Factor-1-Induced Anxiolytic and Antidepressant Effects in a Rat Model of Sporadic Alzheimer’s Disease Are Associated with the Peripheral Suppression of Inflammation
Source: Cells. 2025 Aug 1;14(15):1189. doi: 10.3390/cells14151189 (PMC12346486; doi:10.3390/cells14151189)
Supplement: Supplementary file 1 [file cells-14-01189-s001.zip › cells-3766828-supplementary/Table S3.pdf]

Table S3. The effect of insulin-like growth factor-1 (IGF-1) treatment and time (stage) on the number of red blood cells (RBC), hemoglobin concentration (HGB), mean hemoglobin concentration in the red blood cells (MCHC), mean mass of the hemoglobin in the red blood cells (MCH), mean corpuscular volume (MCV), hematocrit (HCT), red cell distribution width (RDW) (**a**), the number of platelet (PLT), mean platelet volume (MPV), platelecrit (PCT) (**b**) at the early and late stage after intracerebroventricular injections of: streptozotocin and saline (STZ SAL), streptozotocin and insulin-like growth factor-1 (STZ IGF-1), citrate buffer and saline (VEH SAL), citrate buffer and insulin-like growth factor-1 (VEH IGF-1) in the peripheral blood.

a.	**Parameter**	**STZ SAL**	**STZ IGF-1**	**VEH SAL**	**VEH IGF-1**	**STZ SAL**	**STZ IGF-1**	**VEH SAL**	**VEH IGF-1**
	Phase	EARLY STAGE				LATE STAGE			
	RBC (No.x10^6/µl)	7.86±0.56	7.94±0.57	8.23±0.45	8.32±0.47	$7.43\pm0.66^{\$}$	7.72±0.44	8.11±0.48	7.92±0.32
	HGB (g/dL)	14.65±0.39	14.18±0.86	14.65±0.33	14.42±0.43	14.31±0.54	13.91±0.76	14.31±0.57	$13.88\pm0.40^{\$\&}$
	MCHC (g/dL)	33.5±2.27	31.88±0.65	33.02±2.49	32.17±2.41	34.2±1.28	32.91±1.92	32.66±2.11	33.14±1.59
	MCH (pg)	18.75±1.67	18.1±0.81	17.88±1.16	17.37±0.75	19.4±2.03	18.08±0.94	17.7±1.26	17.55±0.73
	MCV (μm^3)	55.75±1.08	$56.58\pm1.43^{\wedge}$	55.17±1.94	54.17±0.98	56.43±4.93	$54.88\pm1.46^{\wedge\&}$	54.14±1.21	52.88±1.13
	HCT (%)	43.83±2.43	44.69±2.47	45.13±2.80	45.27±3.29	$41.83\pm0.76^{\$}$	42.31±1.79	43.9±2.27	$41.93\pm1.65^{\&}$

	RDW (%)	13.8±0.64	13.69±0.52	13.93±0.36	14.38±0.61	14.17±1.55	13.78±0.83	13.43±0.97	14.2±0.77
b.	PLT (No. x10³/µl)	411.25±250.29	376.75±123.46$^	203.50±84.94	219±86.33	175.57±64.17	419.13±118.24<br>**$$^^	203.43±90.33	225.13±106.04
	MPV (µm³)	9.05±2.57	7.8±1.06	8.05±0.50	9.15±2.43	8.16±2.61	8.1±0.58	8.53±1.44	8.16±1.57
	PCT (%)	0.29±0.24	0.28±0.10^^	0.19±0.11	0.11±0.04	0.08±0.05$$	0.36±0.06<br>***$$$^^^	0.17±0.06	0.12±0.05

Explanations: **-p<0.01, ***-p<0.001 indicate significance of differences between STZ SAL and STZ IGF-1, $-p<0.05, $$-p<0.01, $$$-p<0.001 indicate significance of differences to VEH SAL, ^-p<0.05, ^^-p<0.01, ^^^-p<0.001 indicate significance of differences between STZ IGF-1 and VEH IGF-1, &-<0.05 indicates significance of differences between early and late stage of disease progression.

Results descriptions: As shown in Table S3a, at the early phase of sAD, in STZ IGF-1 animals the mean corpuscular volume was higher than in VEH IGF-1 rats (p<0.05). At the late phase, RBC number was lower in STZ SAL rats than in VEH SAL rodents (p<0.05). In VEH IGF-1 group the hemoglobin concentration was lower compared to VEH SAL animals at the late stage (p<0.05). Moreover, HGB in VEH IGF-1 group was lower at the late stage than at the early stage(p<0.05). In addition, in STZ IGF-1 group MCV was higher compared to VEH IGF-1 rats at the late stage (p<0.05). What's more in STZ IGF-1 rats, MCV at the late stage was lower than at the early stage (p<0.05). At the late stage, HCT was lower in STZ SAL rats than VEH SAL animals (p<0.05). In VEH IGF-1 group HCT was lower at the late phase compared to the early stage (p<0.05).

Table S3b shows the results of the platelet system parameters. The number of platelet cells was higher in STZ IGF-1 group compared to VEH SAL and VEH IGF-1 animals (in both comparisons p<0.05) at the early phase. At the late phase, the number of PLT also was higher in STZ IGF-1 group compared to STZ SAL, VEH SAL and VEH IGF-1 rats (in all comparisons p<0.01). The platelecrit was higher in STZ IGF-1 group than in VEH IGF-1 rats (p<0.01) at the early phase of sAD progression. At the late phase the platelecrit was higher in STZ IGF-1 rats than in STZ SAL, VEH SAL, and VEH IGF-1 animals (in all comparison p<0.001). Moreover, PCT was higher in VEH SAL group than in STZ SAL animals at the late stage (p<0.01).
